# Split Ring Resonator Network and Diffused Sensing Element Embedded in a Concrete Beam for Structural Health Monitoring

**DOI:** 10.3390/s22176398

**Published:** 2022-08-25

**Authors:** Erika Pittella, Raissa Schiavoni, Giuseppina Monti, Antonio Masciullo, Marco Scarpetta, Andrea Cataldo, Emanuele Piuzzi

**Affiliations:** 1 Department of Information Engineering, Electronics and Telecommunications (DIET), Sapienza University of Rome, 00184 Roma, Italy; 2 Department of Engineering for Innovation, Complesso Ecotekne-Corpo O, University of Salento, 73100 Lecce, Italy; 3 Department of Electrical and Information Engineering, Politecnico di Bari, Via E. Orabona 4, 70125 Bari, Italy

**Keywords:** structural health monitoring, sensing element, split ring resonator, continuous monitoring, sensor network

## Abstract

The aim of this work is to propose two different and integrated sensors for the structural health monitoring of concrete beams. In particular, a diffused sensing element and a split ring resonator network are presented. The first sensor is able to detect the variations in the dielectric properties of the concrete along the whole beam length, for a diffuse monitoring both during the important concrete curing phase and also for the entire life cycle of the concrete beams. The resonators instead work punctually, in their surroundings, allowing an accurate evaluation of the permittivity both during the drying phase and after. This allows the continuous monitoring of any presence of water both inside the concrete beam and at points that can be critical, in the case of beams in dams, bridges or in any case subject to a strong presence of water which could lead to deterioration, or worse, cause serious accidents. Moreover, the punctual sensors are able to detect the presence of cracks in the structure and to localize them.

## 1. Introduction

Civil infrastructure systems such as bridges, buildings, dams, and pipelines play a central role in the economic and industrial wealth of society [1]. Monitoring the health condition of the above-mentioned infrastructures, often exposed to various external loads and problematic environmental conditions, is of crucial importance to avoid failures and for planning maintenance [2]. For these reasons Structural Health Monitoring (SHM), which includes the integration of sensors, smart materials, data transmission, computational power, and processing ability integrated within the structures [3], is currently one of the leading research topics of structural engineering [4].

The most widely used construction material in the world is the concrete, thanks to its strength, durability, versatility, and cost effectiveness. Concrete is a mixture of Portland cement, water, aggregates, and in some cases, admixtures; its properties depend on the relative amounts and characteristics of the individual components. Even a perfect mix can result in poor quality concrete if the curing phase is not properly monitored [5]. The concrete hardening phase period is considered complete within the first 28 days (with the first 48 h being the most critical) during which an accurate monitoring is of vital importance [6].

Indeed, a premature removal of formworks may lead to inadequate structure strength and to the presence of cracks, produced by internal possible strain, that can cause the collapse of the structure at a later time. Moreover, the ex-post monitoring also has great importance, since it allows an immediate response in case of anomalies, decay phenomena, and also in the case of structures in contact with water such as dams, riverbanks, etc.

Recently, the monitoring of moisture content in concrete structures and their consequent deterioration has triggered the interest in electromagnetic sensors [7,8,9,10].

In particular, in one study [10], the design of a split ring resonator (SRR) network, composed of sensors with different resonance frequencies to both monitor and localize possible cracks, was proposed. Preliminary experimental results showed that the sensors can monitor a concrete crack of few millimeters. These sensors can offer an accurate but relatively confined monitoring of the structure; therefore, the combination with a wire-like diffused sensing element [11] can be a winning solution in order to monitor the structure along the entire length, for detecting dielectric variation both during the curing process and for the entire useful life of the structure.

On the bases of the results presented in [10], the number of SRRs has been extended and the network has been designed, fabricated and tested in a realistic scenario, namely integrated in a concrete beam together with a wire-like diffused sensing element. Both sensors can be permanently embedded in buildings, structures, infrastructures at the time of construction, returning a response based on material dielectric characteristics, particularly useful for early identification of destructive phenomena.

The present work is organized as follows. Section 2 presents the SRR network design and the diffused sensing element. Section 3 describes the experimental set-up. Experiment results are reported in Section 4. Finally, conclusions and the future developments are outlined in Section 5.

## 2. Split Ring Resonator Network Design

### 2.1. SRR Network

An SRR is made up of metal tracks on a dielectric substrate; these tracks consist of circular crowns with cuts along a diameter, hence the name “Split Ring” resonator. There are various types of SRR which differ in geometry [12]. Starting from microwave applications of SRR [13,14], we focused on SRRs used for material dielectric characterization [15,16,17] and, in particular, SRRs with high sensitivity, high quality factor, small size, and that do not require a particular sample preparation [13,18]. As detailed in [10] the choice of the substrate depends on resonator requirements in terms of quality factor, possible dispersion effects, and other practical needs as well as availability on the market.

In particular, the substrate thickness affects both the losses and the practical needs for measurement. For the latter, it is obvious that the greater the thickness, the greater the strength of the resonator leading to a good repeatability of the measurements and, especially, to a high accuracy. To determine the suitable substrate and to optimize the geometric parameters of the SRR with reference to the desired resonance frequency and optimal quality factor, parametric simulations were performed with EM CAD CST Microwave Studio [19], using various substrates available on the market and obtaining the scattering parameter matrix for each SRR. These optimization simulations were performed with the SRR in air, employing perfectly matched layer (PML) absorbing boundary conditions, and the datasheet specifications for the substrate material properties. From the analysis of the transmission coefficient and the quality factor, the AD255C material from Rogers Corporation with thickness 0.040” (1.016 mm) was chosen, with ε_r_ = 2.55; loss tangent = 0.0013 @ 10 GHz; t = 0.035 mm, where t is the thickness of the copper cladding [20].

**Figure 1:**
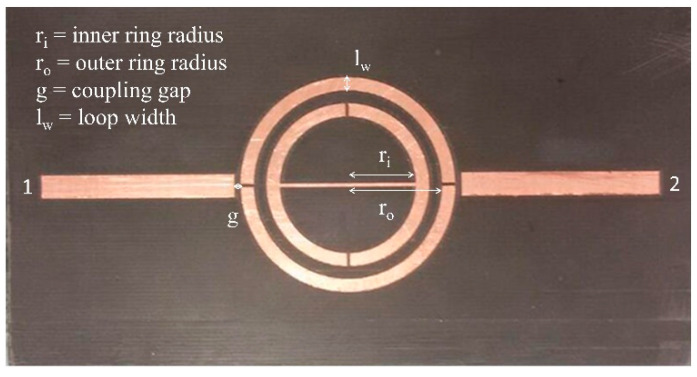
Split ring resonator geometry and its principal parameters.

Four similar SRRs have been designed and optimized, using the same material and varying the geometric characteristics (their principal parameters are displayed in Figure 1) to achieve different resonance frequencies. In this way, a network to be used to monitor concrete structures can be implemented, in order to detect changes in the dielectric properties of the material under test and/or to identify the presence of possible cracks in the vicinity of the sensitive elements.

**Figure 2:**
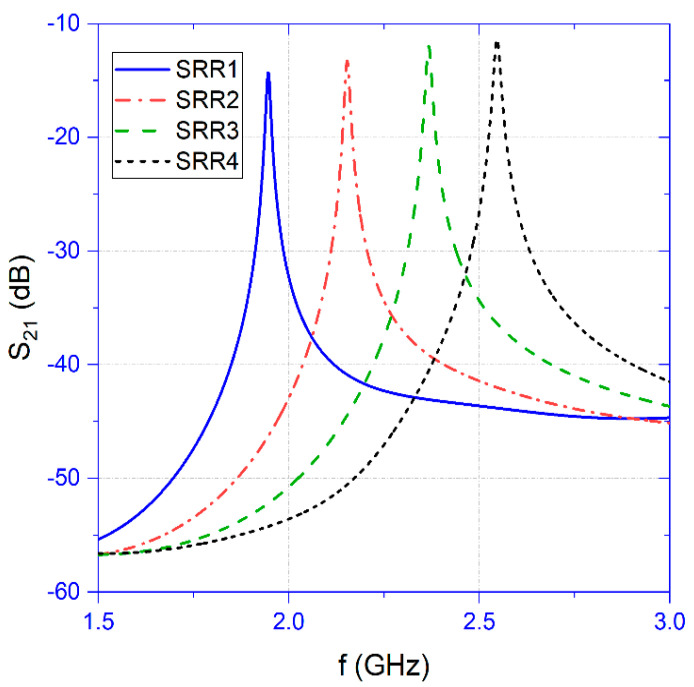
Transmission coefficients of split ring resonators in air.

Table 1 shows the geometrical characteristics for the four SRRs and Figure 2 shows the corresponding transmission coefficients (scattering parameter S_21_) for the four sensors operating in air.

**Figure 3:**
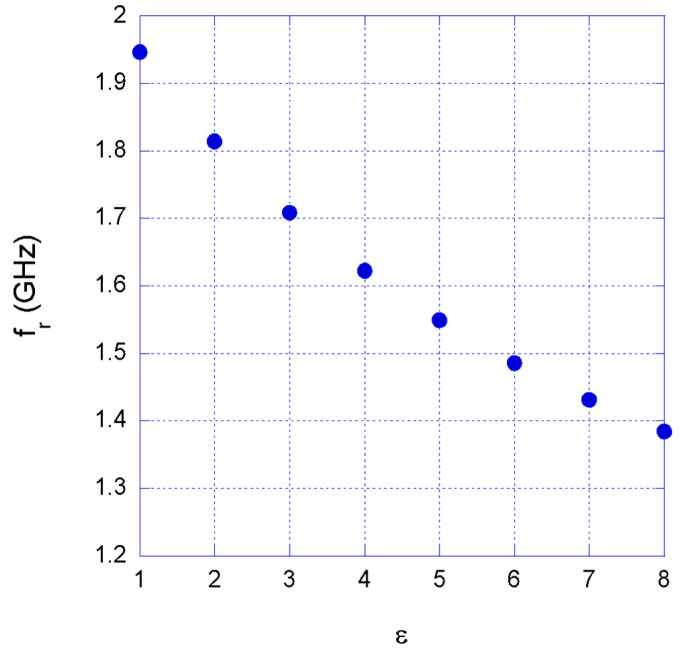
Relationship between the SRR1 resonance frequency and the permittivity of the surrounding material.

Subsequently, the SRRs were simulated with the numerical CAD, embedded in a medium with a permittivity ε that has been varied from 1 to 8 in step 1. Figure 3 shows the relationship between the resonance frequency *f_r_* and the permittivity ε for the SRR1, while Table 2 contains simulation results for all four SRRs.

### 2.2. Power Divider

To feed the four SRRs, the design of a power divider has been conducted with Microwave Office (MWO) by AWR [21]. In particular, a resistive power divider is considered since it is the simplest circuit topology, the smallest, and the most broadband; on the other hand, it also has high losses.

**Figure 4:**
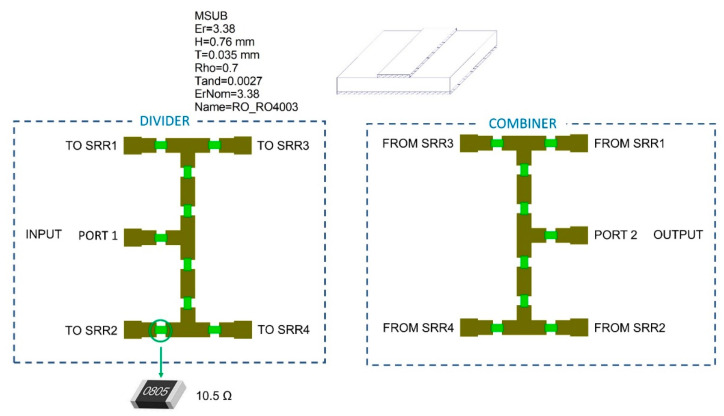
Scheme of the network with the MWO schematic of the power divider and combiner.

Figure 4 shows a schematic of the network: the presence of identical 16 × 2/3 Ω resistors ensures that the divider does present an input impedance of 50 Ω at all the ports [22]. The substrate used for the circuit design is the Rogers RO4003, whose principal characteristics are contained in Figure 4. The same scheme has been adopted for the combiner, that is used at the SRRs output to combine the four-feed lines into a single one, to allow transmission coefficient measurements of the entire network. The MWO simulation results for the divider/combiner network highlight an insertion loss ranging from 10 to 15 dB within the SRR operating frequency band. The scattering parameters of the single SRRs obtained through the CST full-wave simulations were used for implementing the entire sensor network within MWO software, thus obtaining the S_21_ of the entire sensor network.

**Figure 5:**
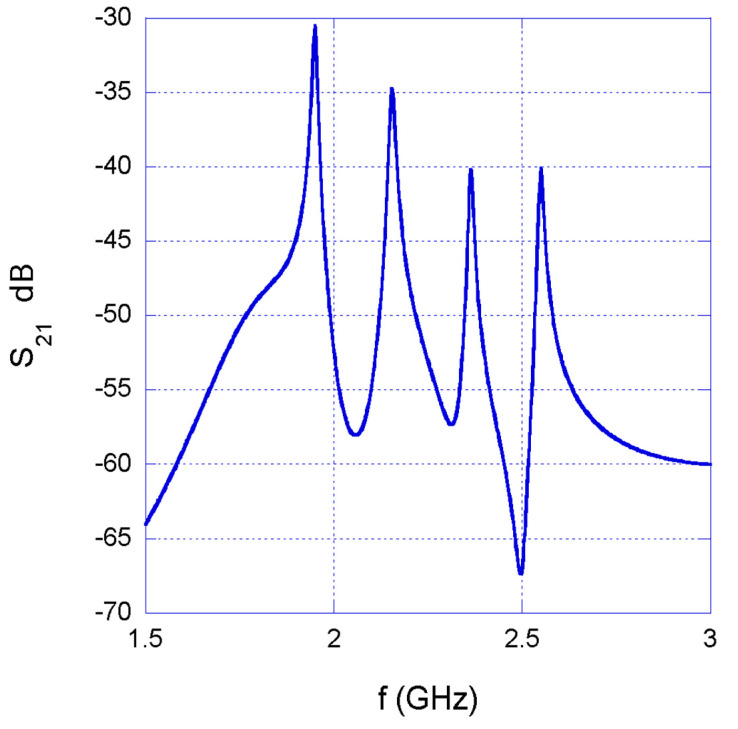
S_21_ MWO simulation of the designed network.

Figure 5 shows the S_21_ of the entire sensor network (ports 1 and 2 are the input and output ports, respectively, as shown in Figure 4) in the case of SRRs embedded in a material equal to air, i.e., with ε = 1. The simulation shows four peaks in the S_21_ response of the entire network which, as expected, correspond to the resonance peaks of the four SRRs previously designed. Comparing Figure 2, which refers to the single SRRs in air, and Figure 5, which refers to the entire network in similar conditions, a decrease of 20 to 30 dB in transmission can be observed, which is due to the previously highlighted insertion losses of the power splitter and combiner.

### 2.3. Permittivity Detection through SRR Network

For the purpose of the present work, it is important to be able to evaluate the concrete permittivity starting from the SRR network response. This allows, in the first place, monitoring the concrete status during the curing phase. Furthermore, exploiting the same network embedded in the concrete structure it is possible to implement practically a continuous health monitoring.

All this information can be directly related to the changes in the resonance frequencies of the single SRRs as a result of variations in concrete permittivity. These variations can result from different factors, such as moisture content or air gaps resulting from concrete deterioration. For this purpose, as already highlighted in Section 2.1, specific CST parametric simulations were performed for each SRR changing the permittivity of the surrounding medium (background material) and evaluating the corresponding changes in the scattering matrix.

In this way, calibration curves relating resonance frequency (*f_r_*) to material permittivity (ε) can be obtained. As reported in the literature [13], a second-order polynomial fitting is usually suitable for accurately describing the variations in resonance frequency as a function of permittivity:

(1) $$\varepsilon \left(f_{r}\right)=p_{1}f_{r}^{2}+p_{2}f_{r}+p_{3}$$

where:

$$p_{1}=13;\;\;\;\;p_{2}=-55.5;\;\;\;\;p_{3}=59.84\;\mathrm{for}\;\mathrm{SRR}1\;p_{1}=10;\;\;\;\;p_{2}=-47.68;\;\;\;\;p_{3}=57.34\;\mathrm{for}\;\mathrm{SRR}2\;p_{1}=8.244;\;\;\;p_{2}=-43.26;\;\;\;\;p_{3}=57.27\;\mathrm{for}\;\mathrm{SRR}3\;p_{1}=7.067;\;\;\;p_{2}=-40.02;\;\;\;\;p_{3}=57.12\;\mathrm{for}\;\mathrm{SRR}4.$$

In this way, starting from the resonance frequency of the SRR and applying Equation (1), the corresponding permittivity of the material surrounding the sensor can be obtained.

### 2.4. Wire-Like Diffused Sensing Element

The diffused sensing element consists of two cylindrical conductors, parallel to each other and mutually isolated by a plastic covering. This type of sensor, embedded in the system under test (SUT) has one end accessible to perform measurements, allowing a diffused monitoring of SUT and providing information on the entire profile of the structure.

In particular, the microwave reflectometry electromagnetic (EM) measurement technique is used, employed in many monitoring and diagnostic applications [11,23]. An EM signal propagates through the sensing element located in the SUT. By analyzing the signal that is partially reflected toward the measuring instrument, it is possible to obtain the wanted information on the SUT, such as for example water content, structural damage and so on.

**Figure 6:**
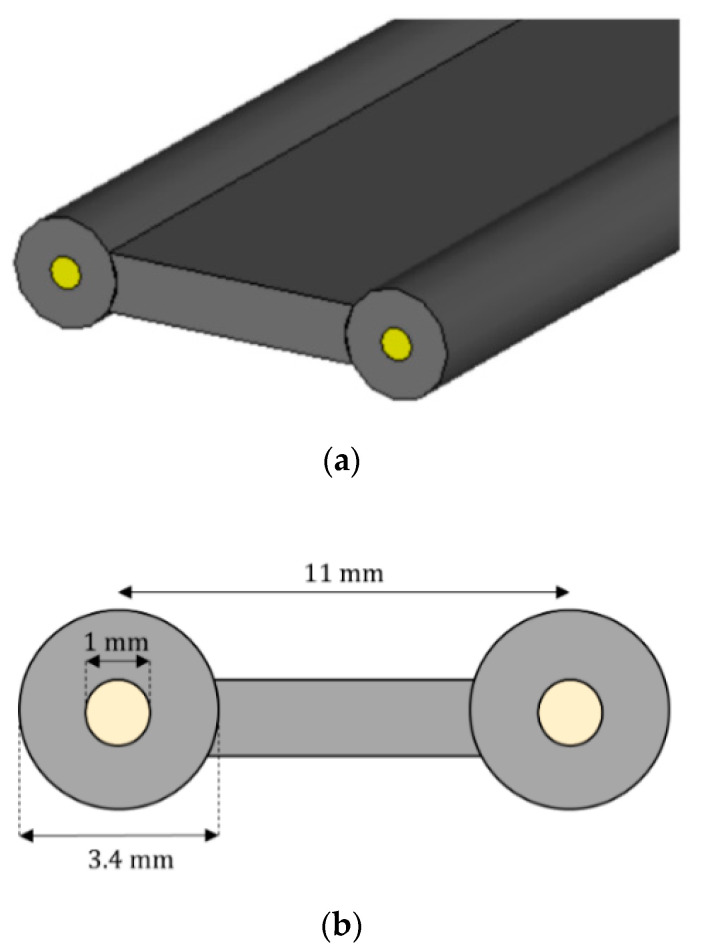
Sensing element perspective view (**a**) and cross section (**b**).

Figure 6 shows a scheme of the employed sensing element.

The EM signal, usually a step-like signal, propagates along the sensing element and whenever it encounters an impedance variation, linked to the changes in the dielectric characteristics of the SUT, a partial reflection of the signal is generated. The TDR instrument acquires the reflection coefficient ρ, that is displayed as a function of time or the traveled apparent distance d_app_ (the distance the signal would travel, in the same time interval, if it was propagating in vacuum); it is given by:

(2) $$\rho =\frac{v_{r}\left(t\right)}{v_{inc}\left(t\right)}$$

where, *v_r_* (*t*) is the amplitude of the reflected signal and *v_inc_* (*t*) is the amplitude of the incident signal.

From the sensing element physical length (*L_r_*) the material effective dielectric constant can be achieved estimating the apparent length (*L_a_*) from the reflectogram:

(3) $$\varepsilon_{app}=2\left(\frac{L_{a}}{L_{r}}\right)$$

considering that the propagation velocity of the signal inside the propagation medium depends on material dielectric properties ε*_app_*, and reflects the interaction between the EM signal and the SUT.

The sensing element scheme of Figure 6 shows the two sensor wires [24].

## 3. Experimental Set-Up

**Figure 7:**
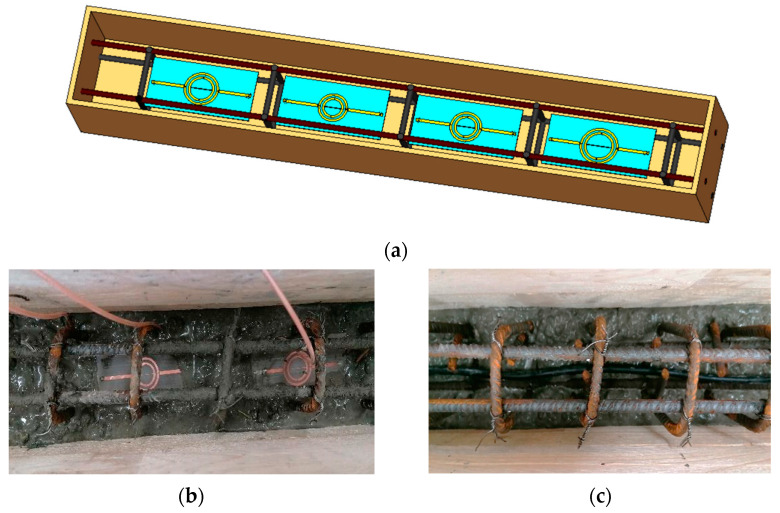
Scheme of the experimental setup. (**a**) 3-D view of the four SRRs embedded in the concrete beam. (**b**) Picture of the setup showing a detail of the top portion of the beam housing two SRRs during concrete pouring in the formwork. (**c**) Picture showing a detail of the bottom portion of the beam housing the diffused SE.

The SRR network and the sensing element were embedded in a concrete mix inside a formwork with dimensions 1 m × 0.15 m × 0.15 m. The divider, the combiner and the four SRRs were assembled together, connecting them with flexible coaxial cables. In particular, during the concrete pouring inside the formwork, a first layer of concrete, inside which the diffused sensing element has been embedded, was deposited in the bottom. Subsequently, a second layer of concrete, housing the SRR network, was poured in the upper part of the formwork. The two concrete layers were prepared with a slightly different mixture composition, with a higher cement-to-aggregate ratio in the bottom layer. This different mixture was intentionally created to achieve a lower permittivity around the four SRRs in order to test the effective localization capacity of the variation of the material in the surrounding area. Figure 7 shows the experimental set-up; (a) 3-D view of the four SRRs realizing the distributed network embedded in the concrete beam, (b) a detail of two SRRs inside the formwork during concrete pouring, (c) detail of the SE. This configuration of the network can provide accurate data on four different zones, while the sensing element can supply a diffused monitoring of the system.

A miniaturized vector network analyzer (VNA), namely the nanoVNA (Amsterdam, The Netherlands) is employed for frequency domain measurements. The nanoVNA is a compact miniaturized VNA, with dimensions 15 cm × 10 cm × 6 cm, it is low-cost and it has 50 kHz–3 GHz frequency range. It was connected to the four split ring resonators through the power divider and combiner allowing the network S_21_ measurement.

For the TDR measurements a Campbell-Scientific TDR200 (Leicestershire, UK) is used, a low-cost, portable TDR measuring instrument, with dimensions 22 cm × 5 cm × 11 cm. The TDR200 generates a step-like voltage signal, with a 200 ps rise time, which corresponds to a frequency bandwidth of approximately 1.7 GHz, that propagates through the sensing element embedded in the concrete beam [25,26].

In this way, measurements were performed over a 28-day period, every day, allowing an accurate monitoring of the concrete curing phase.

## 4. Results

### 4.1. Measurements in Air

**Figure 8:**
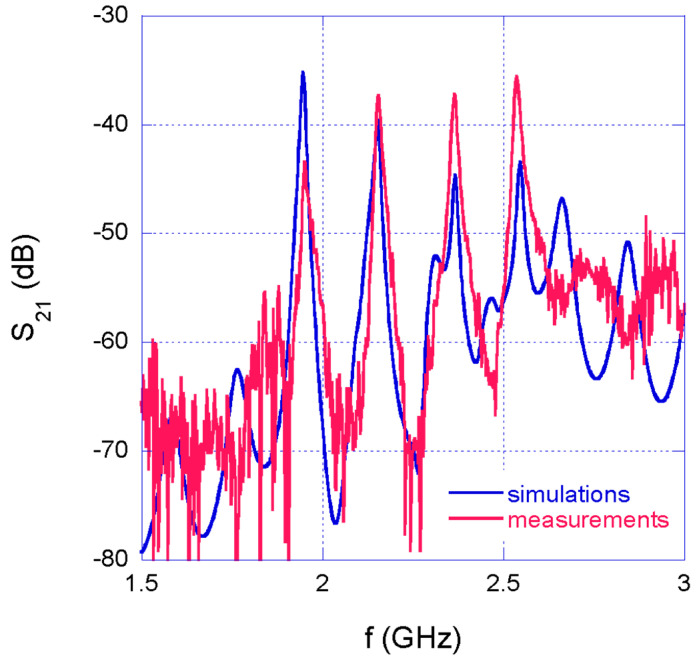
Measured and simulated network transmission coefficient in air.

First, the network transmission coefficient measurements were carried out though the nanoVNA in air, before embedding the network in the concrete beam. Figure 8 shows the measurement results compared with simulations. The experimental curve shows, as expected, large noise levels below −60 dB, due to the intrinsic noise figure of the low-cost miniaturized VNA; however, this noise floor does not affect measurements around the resonance peaks, which occur at much higher levels. It is worth noting here that the MWO circuit model was improved, in order to obtain the same experimental conditions, by adding to the original circuit schematic the coaxial cables used for connecting the SRRs to the combiner and divider. Due to the presence of the cables, multiple reflections between the SRRs and the combiner/divider networks occur giving rise to additional peaks linked to the resulting stationary waves. These additional peaks are visible on the network transmission coefficient, but they do not affect the detection of the resonance peaks, which are still clearly distinguishable, both in simulation and measurement results. Finally, the observed deviation in amplitude between simulated and experimental data are caused by losses resulting from parasitic effects and non-idealities intrinsically introduced by the experimental setup. However, the results in Figure 8 clearly show an excellent agreement between experimental and simulated data with reference to resonance frequencies, which are the most significant parameters related to the sensing task.

### 4.2. Measurements in the Concrete Beam

After inserting the four SRRs and the sensing element inside the beam during the concrete pouring, measurements on both types of sensors were repeated during the 28 days of hardening of the concrete.

As regards the SRRs, the first day, the presence of a large quantity of water produced a S_21_ which looks like a large bell in which the peaks merged together due to the high losses dramatically decreasing the quality factor. On the second day, however, much of the water has evaporated and the four peaks begin to be clearly visible and shift to the right, as expected when permittivity begins to drop.

**Figure 9:**
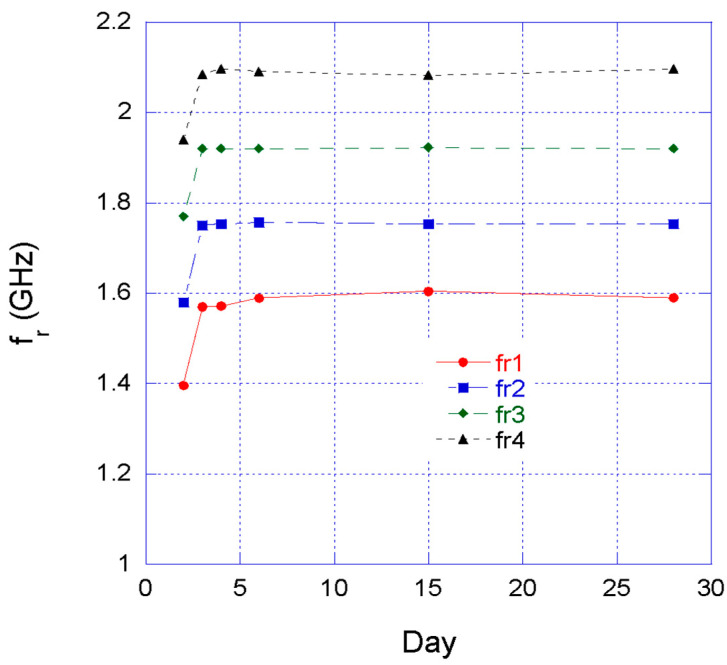
Computed network transmission coefficient resonance frequency during the curing phase.

Figure 9 shows the resonance frequency measured during the curing phase; in particular, the 2nd, 3rd, 4th; 6th, 15th and 28th days are displayed. The resonance frequency of the four SRRs were extrapolated by fitting the data with a Lorentzian curve using MATLAB. In this way, from the resonance frequencies and by applying the calibration curve (1) for the corresponding split ring, the permittivity of the concrete can be extrapolated.

**Figure 10:**
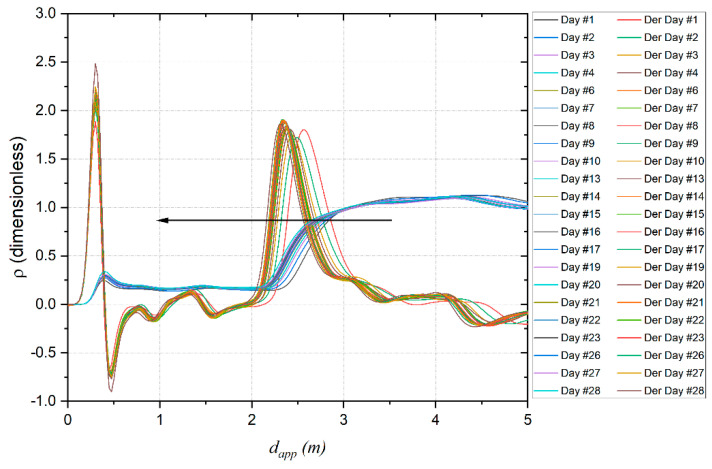
Reflection coefficient as a function of the apparent distance and the related derivative.

On the two-wire sensing element it is noted that as the days pass, the apparent length decreases (see Figure 10). In particular, Figure 10 shows the reflection coefficient as a function of the apparent distance and the related derivative as the days pass.

**Figure 11:**
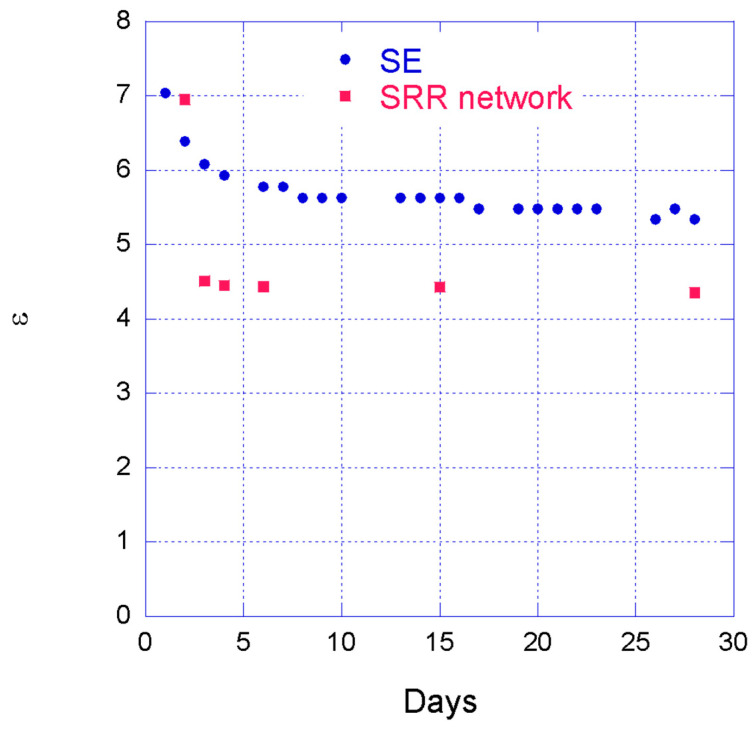
Measured permittivity during the curing of the concrete.

To extrapolate the permittivity of the concrete, the inversion of the measurements in the time domain was carried out using a Matlab algorithm, using a step as an incident pulse. By applying (3) the permittivity can be achieved. Figure 11 shows the obtained results both with the diffused SE and the SRR network.

It is worth noting here that, as already mentioned, the first day the S_21_ has a bell-shaped trend and the four resonances of the SRR network are not clearly visible; this is the reason it was not possible to extrapolate permittivity data with the SRR network in Figure 11 for the first day.

Another important aspect is that the difference in concrete permittivity was deliberately made. In fact, in order to verify if it is possible to notice variations in the surroundings of the planar sensors and therefore to allow precise monitoring, a different mixture of cement was placed around the SRRs in the top layer of the beam structure, with a slightly lower cement-to-aggregate ratio, resulting in a lower permittivity.

Even though the reported experimental results referred only to monitoring of the curing phase, a monitoring of this type will also allow controling the presence of water inside the beam [27,28,29,30,31,32], but also to locate any internal cracks in the beam [10,33] during the entire lifespan of the concrete structure.

## 5. Conclusions

In this paper, two sensors for concrete monitoring were presented, a diffuse SE and a network of SRRs for punctual monitoring. The first sensor is able to monitor both the curing phase and possible anomalies arising during the lifespan (water intrusion, detachment of concrete portions, etc.) of the concrete structure, which might also be several hundreds of meters long. The second sensor typology, instead, is able to monitor both the presence of water inside the beam and any cracks around the sensor and determine its location thanks to the different working frequencies of resonators; however, the SRR network is only able to cover localized portions of the structure so as to achieve a detailed and sensitive monitoring of possible critical sections of the structure with a distributed localized approach.

The integration of these two structures makes it possible both to monitor concrete during the curing phase, which is very important for the future structural strength of the beam, and to highlight the presence of water that could cause deterioration, especially in the cases of dams, bridges and structures that are constantly subjected to an important presence of water.

For future developments it is planned to test the possibility of monitoring the presence of water near one of the SRRs and the localization of artificial cracks, specifically created with mechanical stress in some points of the beam.

Although the present study was limited to a frequency range compatible with portable low-cost instruments, a very interesting future development could regard the adoption of miniaturized THz-band sensing structures, thus allowing for a finer distributed sensor network as well as a more efficient detection on micro-cracks occurring during the lifespan of the concrete structure. Such THz structures were recently proposed in the literature [34,35,36,37,38].

## Figures and Tables

**Table 1:** Geometric dimensions of Split Ring Resonators.

SRR	r_i_ (mm)	r_o_ (mm)	g (mm)	l_w_ (mm)
1	14.0	18.0	0.44	2.0
2	12.4	16.4	0.44	2.0
3	11.0	15.0	0.44	2.0
4	10.0	14.0	0.44	2.0

**Table 2:** Resonance frequency of the four split ring resonators embedded in a material with permittivity ε.

ε	1	2	3	4	5	6	7	8
*f_r,SRR1_*	1.9461	1.8141	1.7082	1.6224	1.5489	1.4856	1.4313	1.3842
*f_r,SRR1_*	2.1531	2.0091	1.8894	1.7910	1.7109	1.6407	1.5771	1.5222
*f_r,SRR3_*	2.3670	2.2101	2.0772	1.9710	1.8819	1.8039	1.7364	1.6737
*f_r,SRR4_*	2.5464	2.3733	2.2347	2.1231	2.0268	1.9431	1.8690	1.8015

## Data Availability

The data presented in this study are available on request from the corresponding author.
